# Toward Repurposing Metformin as a Precision Anti-Cancer Therapy Using Structural Systems Pharmacology

**DOI:** 10.1038/srep20441

**Published:** 2016-02-04

**Authors:** Thomas Hart, Shihab Dider, Weiwei Han, Hua Xu, Zhongming Zhao, Lei Xie

**Affiliations:** 1 The Rockefeller University, New York, New York, United States of America; 2 Department of Biological Sciences, Hunter College, The City University of New York, New York, New York, United States of America; 3 The Key Laboratory for Molecular Enzymology and Engineering, Ministry of Education Jilin University, Changchun, P. R. China; 4 School of Biomedical Informatics, The University of Texas Health Science Center at Houston, Houston, Texas, United States of America; 5 Department of Biomedical Informatics, Vanderbilt University School of Medicine, Nashville, Tennessee, United States of America; 6 Department of Psychiatry, Vanderbilt University School of Medicine, Nashville, Tennessee, United States of America; 7 Department of Cancer Biology, Vanderbilt University School of Medicine, Nashville, Tennessee, United States of America; 8 Center for Precision Health, School of Biomedical Informatics, The University of Texas Health Science Center at Houston, Houston, Texas, United States of America; 9 Ph.D. Program in Computer Science, Biology, and Biochemistry, The Graduate Center, The City University of New York, New York, New York, United States of America; 10 Department of Computer Science, Hunter College, The City University of New York, New York, New York, United States of America

## Abstract

Metformin, a drug prescribed to treat type-2 diabetes, exhibits anti-cancer effects in a portion of patients, but the direct molecular and genetic interactions leading to this pleiotropic effect have not yet been fully explored. To repurpose metformin as a precision anti-cancer therapy, we have developed a novel structural systems pharmacology approach to elucidate metformin’s molecular basis and genetic biomarkers of action. We integrated structural proteome-scale drug target identification with network biology analysis by combining structural genomic, functional genomic, and interactomic data. Through searching the human structural proteome, we identified twenty putative metformin binding targets and their interaction models. We experimentally verified the interactions between metformin and our top-ranked kinase targets. Notably, kinases, particularly SGK1 and EGFR were identified as key molecular targets of metformin. Subsequently, we linked these putative binding targets to genes that do not directly bind to metformin but whose expressions are altered by metformin through protein-protein interactions, and identified network biomarkers of phenotypic response of metformin. The molecular targets and the key nodes in genetic networks are largely consistent with the existing experimental evidence. Their interactions can be affected by the observed cancer mutations. This study will shed new light into repurposing metformin for safe, effective, personalized therapies.

Metformin, a drug frequently prescribed for type 2 diabetes, has been shown to decrease the incidence of cancer in diabetic patients by 37% relative to the diabetic patients not taking this drug, making it an important candidate for drug repurposing^1^. In breast cancer, as well as other cancers, metformin is believed to activate the AMPK signaling pathway, which elicits its anti-cancer effect by inhibiting the mTOR pathway^2^,^3^. In addition to the AMPK pathway, several AMPK independent pathways such as RAS^4^, AKT^2^, and HIF-1α^5^ may contribute to the anti-cancer effect of metformin. Metformin has also been proposed to act directly on mitochondria, decreasing mitochondrial respiration and lowering overall energetic efficiency; in cancer cells, this metabolic stress results in a greater increase in aerobic glycolysis than in normal cells, due to the former’s greater metabolic vulnerability^6^. More recently, Sun *et al.* attempted to construct a signaling pathway network of the genes through which metformin elicits its effect^7^. They proposed that seven genes (*CDKN1A*, *ESR1*, *MAX*, *MYC*, *PPARGC1A*, *SP1*, and *STK11*) and one MYC-centered pathway with *CDKN1A*, *SP1*, and *STK11* might play important roles in metformin’s therapeutic effect. Larsson *et al.* examined the differential influence of metformin on the transcriptome and translatome of cancer cells. Their findings indicate that metformin’s primary effect is of inhibition of translation of a subset of mRNAs^8^. Previously, we proposed that metformin may weakly bind to the AMPKβ subunit, as well as four other proteins which have very similar binding sites (MAP2K2, PDK2, EGFR, and TIAM1)^9^. In spite of these efforts, the molecular and genetic mechanisms of metformin’s anti-cancer effect have not yet been fully understood. First, the direct molecular targets of metformin and their molecular details of interaction, which lead to its pleiotropic effect, are largely unknown. Such information is critical to understand the personalized therapeutic effect of metformin, as it will allow us to predict the direct impact of amino acid mutations on the drug-target interaction. Second, it is not clear what other molecular and genetic factors that modulate the drug action are responsible for the anti-cancer sensitivity of metformin on a patient-specific basis. It is speculated that genetic modifications in the drug modulation pathway will alter the drug response^10^. The answers to these questions will facilitate identifying pharmacogenetic biomarkers for the efficacy and side effects of metformin for individual patients, a critical step towards repurposing metformin as a safe and effective precision anti-cancer therapy.

There is evidence that polypharmacology is a common phenomenon. Weak interactions with many targets make a significant contribution to a drug’s effect^11^. Moreover, cryptic genetic factors may collectively affect drug actions under drug treatments^12^. It is challenging to determine genome-wide drug-target interactions and their correlations with clinical responses under diverse genetic backgrounds both experimentally and computationally. To address this problem, our innovation here is to extract the key proteomic and genomic factors which explain the drug’s empirically-observed activity on a multi-scale, from molecular details of drug-target interactions to the emergent properties of biological networks. Our strategy creates a start-to-finish picture of the drug’s overall effects, from its directly-binding upstream protein targets to the key down-stream proteins which control the body’s anti-cancer machinery. The strategy also takes instances of congruence as suggestions of polypharmacology, producing multiple binding partners and their modulated pathways of metformin’s interaction network. Using only publically available datasets, our methodology has produced a more complete image of metformin’s biological activity. While we have experimentally verified the interaction between metformin and the majority of the targets we have tested, our results have yielded many additional direct and indirect targets ready for further investigation. Our approach is generalizable to any compound with at least one documented protein receptor for which a crystal structure is available and for which a genome-wide gene expression analysis has been performed, and is particularly suited for identification of polypharmacology.

Specifically, we have developed a structure-based method to identify the targets of a drug molecule on a structural proteome scale, and successfully applied it to drug repurposing^13^, side effect prediction^12^, and polypharmacology^14^. Here, a similar strategy will be applied to identify proteome-wide molecular targets of metformin. Among the top ranked targets, several are kinases. Subsequently, we commissioned a KINOMEscan binding assay between these putative kinase targets and metformin, and validated 5 out of 6 our predictions being correct. After identifying putative interacting proteins, we for the first time developed a novel method to predict the downstream genes and drug modulation pathways by integrating drug targets, protein-protein interaction network, and gene expression using a Prize-Collecting Steiner Tree (PCST) algorithm^15^. Finally, we analyzed these predicted networks for biological relevance to anti-cancer activity and to determine hidden genetic factors that will impact the drug modulation pathways. In this way, we can correlate drug-target interactions with drug response phenotypes under diverse genetic backgrounds.

Using the multi-scale modeling approach, we have identified twenty putative targets of metformin. Although using different structural templates as a starting point, the two studies resulted in consistent results. Five kinases that we have shown bind directly to metformin (SGK1, EGFR, CDK7, MAPK14, and MAP2K2). Furthermore, we identified genetic factors (*TP53, AKT1*, and *PCNA* genes) that do not directly bind to metformin, but are responsible for the anti-cancer effect of metformin. Our predictions are largely consistent with the existing experimental and computational results. They generated abundant testable hypotheses for further *in vitro* and *in vivo* validation, and may shed new light into repurposing metformin for safe, effective, personalized anti-cancer therapies. This study also lays the groundwork for high-throughput prediction of pleiotropic drug/target/gene relationships.

## Results

**Figure 1:**
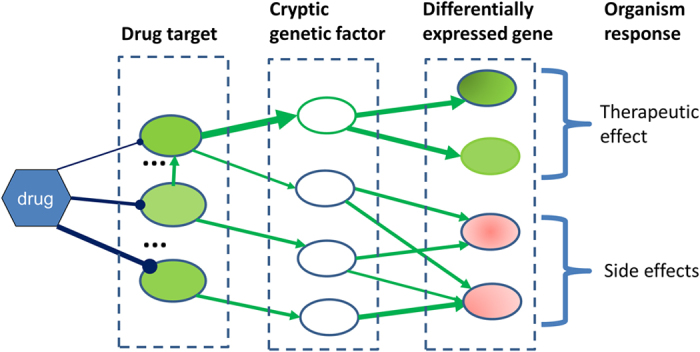
A conceptual framework of structural systems pharmacology used in this study. Dark blue lines represent direct molecular interactions between drug and its targets. Green arrows are protein-protein interactions through which the information of drug-target interaction is transmitted.

**Figure 2:**
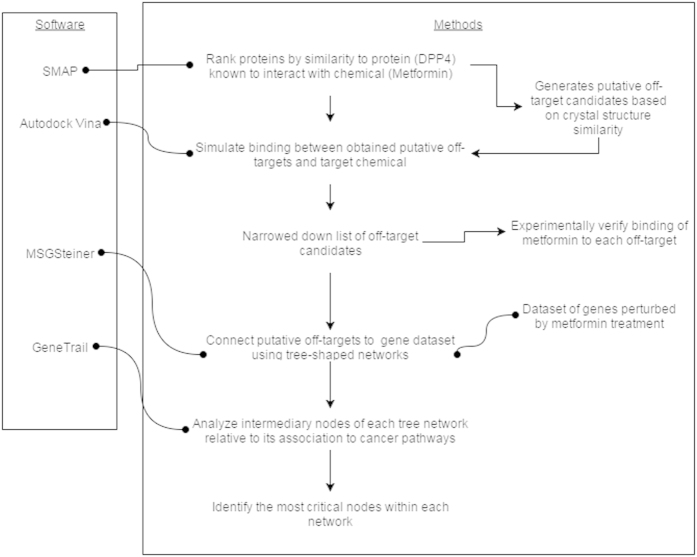
Methodology Flow Chart. Bulleted line segments represent datasets and software used; arrows represent the flow of information and the transition from one step to another.

Our structural systems pharmacology approach is based on a simplified model of drug action as shown in Fig. 1. We assume that a drug like metformin may bind to multiple proteins (drug targets)^12^. Functional modifications of targets resulting from the drug binding (inhibition or activation) will propagate to other genes through a protein-protein functional association network, and manifest in the transcriptional, translational, and other observable genome-wide changes. The observed differentially expressed genes upon drug binding can be used as a surrogate of drug response phenotype. Genes without observable changes (cryptic genetic factors) may exist to mediate the information transmission from drug targets to differential genes. A genetic alteration in either a drug target or a cryptic genetic factor will impact the drug’s action. Following this concept, our structural systems pharmacology approach consists of three major steps. First, we apply a previously-proven successful method to identify drug binding targets on a structural genome scale. Then we map the drug targets and differential genes to a protein-protein interaction (PPI) network and identify the drug modulation pathway, a sub-network on the PPI network, which links the drug targets to differentially expressed genes. Subsequently, we detect the critical gene nodes in the drug modulation pathway whose deletion will have significant impacts on information transmission, thereby altering the drug response phenotype. Finally, we map the observed cancer mutations to the binding pockets of experimentally validated molecular targets and identified genetic biomarkers. These analyses provide clues to the alternation of drug-target interaction and network rewiring that may impact the personalized drug response. The method is summarized in Fig. 2, and details are presented in the Supplemental Methods section.

### Putative targets of metformin

We proposed that metformin might weakly bind to the AMPKβ subunit^9^. DPP4 was identified as another direct molecular target of metformin by querying the ChEMBL relational database using SQL^16^ (Supplemental Methods, Figure S1), and supported by recent studies^17^,^18^. Using AMPKβ subunit and DPP4 as templates, we profiled protein structures from the Protein Data Bank (PDB) to identify those with evolutionarily related and similarly shaped binding pockets to that of AMPKβ and DPP4. We identified 19 structures with a binding-site similarity (multiple testing corrected p-value < 0.05) which were not homologs of AMPKβ and DPP4. This set was termed the “putative targets”. Our top-ranked structure, associated with the Fibroblast Activation Protein (FAP), is a member of the prolyl peptidase protein family – the same family as DPP4, explaining its near-identical binding site^19^. Among the putative targets, six of them are protein kinases.

### Binding mode of metformin in putative targets

**Figure 3:**
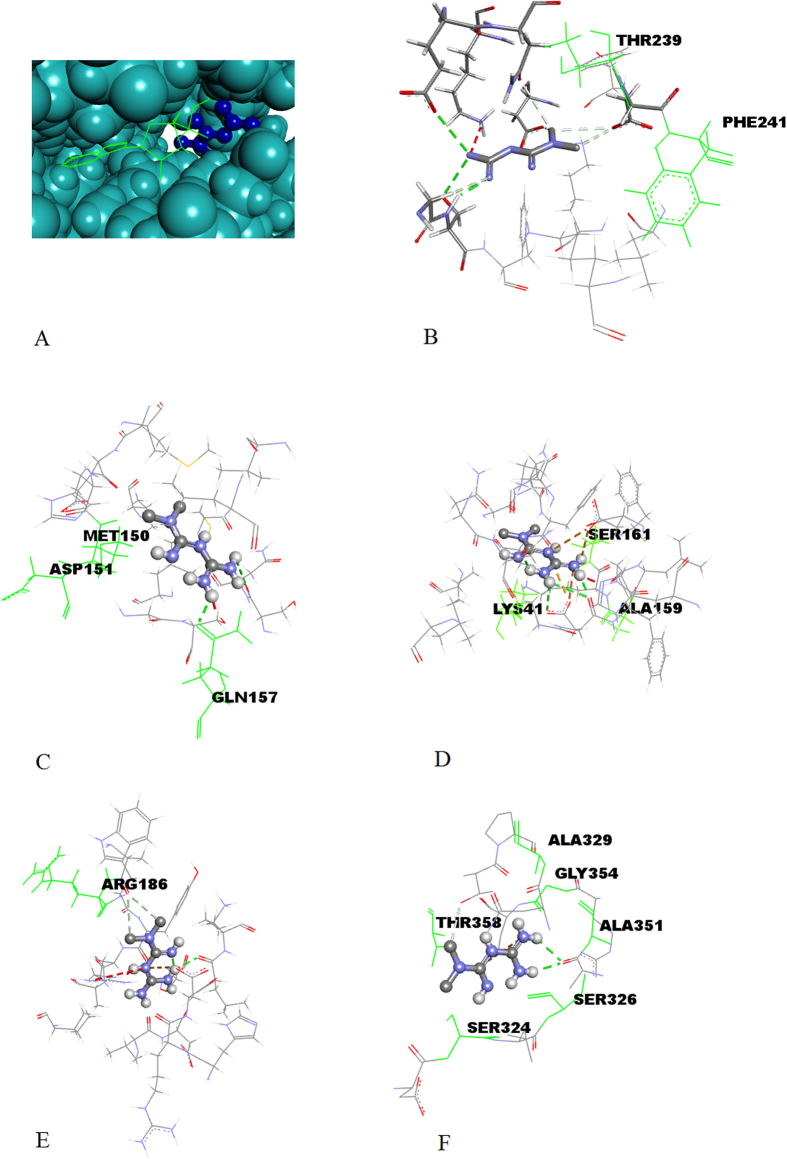
The predicted binding pose of metformin in the binding pocket of putative kinase targets. (**A**) The binding site of metformin (right) is adjacent to the binding site of ATP (left) in SGK1. The interaction patterns of metformin with SGK1 (**B**), MAP2K2 (**C**), CDK7 (**D**), MAPK14 (**E**), and EGFR (**F**). The green, labeled residues are those which are known to be involved in carcinogenic mutations.

Docking model experiments between metformin and each of these proteins predicted exothermic binding interactions to take place in all cases, as shown in Table 1. Figure 3A shows an example of the binding pose of metformin on kinase SGK1. Similar to other kinases, the binding site of metformin is adjacent to the ATP binding site^9^. It implies that metformin may act differently from the common ATP inhibitors of kinases. We further mapped somatic cancer mutations observed in COSMIC (cancer.sanger.ac.uk)^20^ onto the structures of putative kinase targets. A number of these mutations surround the metformin binding site. Five examples are shown in Fig. 3B–F. These amino acid mutations observed in COSMIC are listed in supplementary Table S1. It is speculated that the mutations of these amino acids, will impact the therapeutic effect of metformin. This finding is potentially important in the context of personalized medicine, as metformin may have increased or decreased drug sensitivity in patients with such mutations.

### Binding assay for putative kinase targets

Among our putative targets, six of them are kinases: AKT1, EGFR, MAPK14, MAP2K2, CDK7 and SGK1. We tested the direct binding between metformin and these kinases using

DiscoveRX KINOMEscan^TM^ binding assay. KINOMEscan^TM^ is an active site-directed competition binding assay to quantitatively measure interactions between chemical compounds and kinases. The binding strength is reported by ‘%Control’, which is calculated as shown in equation (1).


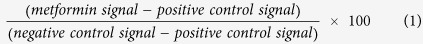


DMSO and a pico-molar kinase inhibitor were used as negative and positive controls, respectively. The lower %Control is, the stronger the interaction. As shown in Table 2, our results showed binding between metformin and all the tested six kinases except AKT1. Of the tested kinases, Metformin showed its strongest binding with SGK1 and EGFR. Thus, our predictions are strongly supported by the experimental evidence.

### Analysis of drug modulated sub-networks

**Figure 4:**
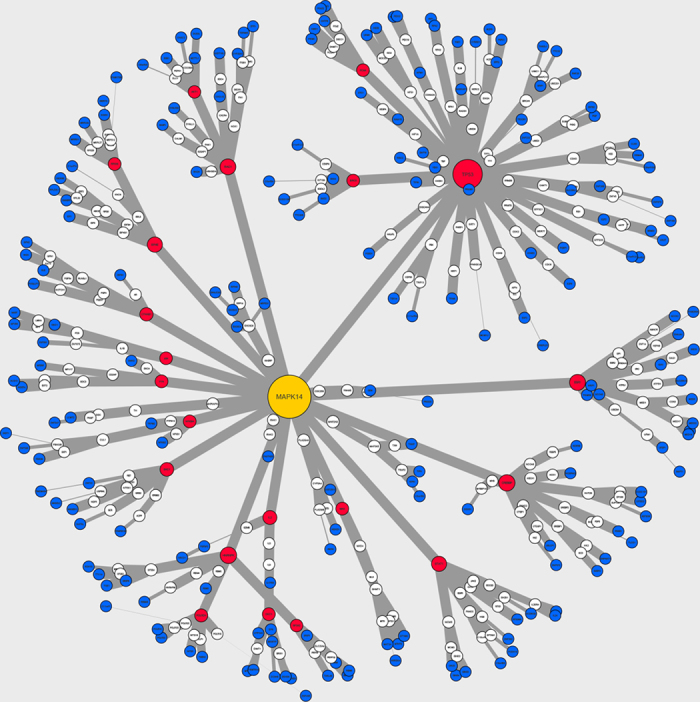
Visualized sub-networks for MAPK14. Blue nodes indicate leaf (differentially-expressed genes); for intermediate nodes (cryptic genetic factors) red indicates critical node; yellow indicates root node; all others are white. Node size correlates with betweenness-centrality score. Edge-width correlates with confidence of the interaction. Note that most interactions are of high relative confidence (>0.9 on a scale from 0.0 to 1.0). The arrangement of nodes in space is for ease of comprehension only. Visualized using CytoScape 3.2.1^54^.

Here, we, for the first time, developed a novel method to predict the drug modulation pathways as protein-protein interaction (PPI) sub-networks, which could lead to experimentally-determined changes in gene expression^8^, using a Prize-Collecting Steiner Tree (PCST) algorithm^15^. In this conceptualization, metformin-interacting proteins form the root of a branching tree network, differentially-expressed genes form the leaves of the tree, and confidence-weighted interactions between other proteins connect the root to the leaves. The PCST algorithm attempts to link the root to as many leaves as possible while minimizing edge cost (maximizing confidence), thus predicting the most linked nodes that take part in the network without apparent alteration of expression. Compared with the conventional shortest path method, the PCST will provide a parsimonious solution that jointly optimizes the paths from the root to all the leaf nodes, thus potentially reducing the occurrence of false positives, especially when applied to the noisy PPI network^11^. In our implementation, protein-protein interactions were obtained from the STRING-DB^21^ and assigned a cost which was calculated as 1 minus the confidence of the interaction. Figure 4 shows an example of the resulting sub-network structure when MAPK14 is chosen as the root. The leaf nodes of our sub-networks were obtained from a set of genes which are translationally-suppressed in MCF7 cells by metformin – as reported by Larsson *et al.*^8^ (see Supplemental Methods). Metformin had been previously demonstrated to inhibit proliferation of MCF7 cells^2^.

We note that the protein-protein interaction scores documented in STRING-DB are noisy. We calculated the distribution of edges both within the STRING-DB human PPI and within our predicted sub-networks. Our results show that the interactions within our networks are consistently of high relative confidence, in contrast to the mainly low relative confidence STRING-DB PPI (Figure S2). This suggests that the PCST algorithm effectively selects high-confidence paths through the STRING-DB PPI and that our predicted sub-networks contain interactions which are likely to occur *in vivo*.

As a control, we generated three additional sets of sub-networks. The first set consisted of sub-networks with randomly chosen genes at the root (the “R-control” set, n = 16). The second set was generated using a random selection of genes from the leaf nodes (“L-control”, n = 20). Noting that our predicted binding targets and binding assay demonstrate the interaction between metformin and several kinases, our third set of control sub-networks were constructed using a random selection of genes coding for kinases (“K-control” set, n = 19).

We used the GeneTrail tool to identify biological pathways that were enriched in the non-terminal nodes of the experimental and control sub-networks. The experimental sub-networks were enriched for a greater number of overall KEGG pathways than the L-control and R-control sub-networks but not the K-control group (average of 40.4, 33.9, 32.8, and 39.4 KEGG pathways respectively; exp vs. L-control < 0.05; exp vs. R-control p < 0.05; exp vs. K-control p = 0.741). The same pattern was seen when comparing enrichment of cancer-related KEGG pathways (average of 22.7, 20.0, 19.5, and 22.8 KEGG pathways respectively; exp vs. L-control p < 0.01; exp vs. R-control p < 0.01; exp vs. K-control p = 0.944). Cancer-related KEGG pathways were defined as pathways related to specific cancer types, e.g. ‘Melanoma’ or ‘Small cell lung cancer,’ as well as pathways known to be involved in the development or maintenance of cancer, e.g. ‘VEGF signaling pathway’ or ‘Apoptosis.’ A full list of KEGG enriched pathways defined as cancer-related can be found in the Supplemental Methods.

The results yielded enrichment of multiple relevant KEGG pathways^22^. ‘Apoptosis’, ‘Cell cycle’, ‘Pathways in cancer’, ‘Chronic myeloid leukemia’, ‘Pancreatic cancer’, ‘Glioma’, ‘Non-small cell lung cancer’, ‘Prostate cancer’, and ‘Bladder cancer’ were all enriched in at least 85% of the sub-networks in each set (at least 18 of 21 experimental sub-networks, 17 of 20 L-control sub-networks, 14 of 16 R-control sub-networks, and 17 of 19 K-control sub-networks, FDR corrected p values < 0.05). Other relevant pathways, including ‘MAPK signaling pathway’, ‘ErbB signaling pathway’, ‘Nucleotide excision repair’, ‘DNA replication’, ‘B cell receptor signaling pathway’, ‘mTOR signaling pathway’, and ‘VEGF signaling pathway’ as well as pathways for several more cancers, were enriched in at least 10 experimental sub-networks and control sub-networks.

As the enrichment of relevant pathways was high in experimental and control sub-networks, we sought a finer method to distinguish between the identified sub-networks.

Based on our KEGG results, as well as our knowledge of metformin’s relevance to cancer, insulin, and AMPK signaling, we assembled a set of ‘key pathways’ which represent a profile of metformin’s documented and putative activity (see Supplemental Methods)^22^. We calculated the number of instances in which nodes of each sub-network were found in a key pathway, and each sub-network was assigned a “participation” score for each key pathway. Although most networks in both the experimental and control groups were enriched for the same pathways, the nodes of the control sub-networks participated less in key pathways. Analysis of participation within key pathways for the sub-networks between the experimental and control groups showed significant differences. On average, the participation of intermediate nodes in key pathways was 117.3 for the L-control, 117.6 for the R-control, 133.7 for the K-control group, and 131.4 for the experimental group. With approximately equal standard deviations of 12.9, 15.6, 13.3, and 18.8, respectively, the difference between experimental and both L-control and R-control is significant (Welch’s T-test for unequal variance, p < 0.01 and p < 0.05 respectively) but the difference between experimental and K-control is not (p = 0.669). Noting that 6 out of 20 of our putative targets were themselves kinases, we calculated that the average participation score for that sub-group (140.8) exceeded that of the K-control, although the difference was not significant (p = 0.416).

The participation scores for experimental sub-networks were normalized against the mean participation the R-control group. This Z-score represents the extent to which the sub-network in question participated in key networks beyond what which we would expect by random chance (Table 3).

In summary, sub-networks generated from randomly-selected genes are not different from sub-networks generated from a random sub-set of the differentially-expressed genes in terms of pharmaceutically relevant pathways. Sub-networks based on the putative targets are significantly more closely related to pathways than either of these sub-network sets. However, there is no observed difference between the putative targets and kinases. Considering the fact that a large number of kinases were experimentally validated targets and that kinases share similar ATP binding pockets, it is likely that many other kinases could be potential targets of metformin.

### Critical components that may impact drug actions

We further determine hidden genetic factors that would impact the drug modulation pathways. Our working hypothesis is that the node in the network would have a greater impact on the drug’s activity if its deletion significantly reduced the flow of information. Table 4 shows genes which appeared as critical nodes (betweenness-centrality in the 95^th^ percentile, see Supplemental Methods^23^,^24^) in at least 20% of experimental sub-networks. We note that several genes were found amongst the critical nodes in at least 50% of sub-networks, in both the set of putative targets and the three control groups. These genes (*TP53*, *PCNA*, *AKT1*, *SRC*, and *INS-IGF2*) were apparently selected because of their functional link to significant sub-sets of the genes which were differentially expressed under metformin treatment^8^, which overwhelmed the choice of root node in the sub-network.

We proposed that because sub-networks with higher Z-scores could better reflect the documented and proposed activity of metformin, they might be closer to metformin’s true biological activity. Because of this, we suggested that nodes showing a higher degree of betweenness-centrality in networks with higher Z-scores in contrast to networks with lower Z-scores should be more likely to be important factors. We segregated the experimental sub-networks based on Z-score into several overlapping sets: all sub-networks, those with a Z-score greater than 1, and those with a Z-score greater than 2. In Table 5 we recorded the genes that have an especially large discrepancy in their betweenness-centrality in different groups of sub-network. For example, *ESR1* is considered critical in 25.0% of R-control sub-networks, 38.10% of all experimental sub-networks, 50.0% of sub-networks with Z > 1, and 80% of sub-networks with a Z > 2. Genes were included in the table if the discrepancy was at least 25 percentage points between highest and lowest percentage.

### Cross-validation of critical components

Garnett *et al.* carried out an elastic-net regression to identify genomic features linked to sensitivity to metformin^25^. Their analysis identified copy number variation in *BRCA1* and *BLM*, two genes found to be critical nodes in our sub-networks, as factors related to sensitivity to metformin in cancer tissue. Expression levels in several other genes were identified through elastic-net regression whose familial counterparts were predicted in at least one sub-network. These include the *IL2RG/IL2, RBM15/RBM5, RPS6KC1/RPS6/RPS16* and *UBE2G1/UBE2I* sets, as well as *MAP4K3* which is related to several predicted *MAP* and *MAPK* nodes and direct targets. Finally, the *AKT3* gene was identified, pairing with the predicted *AKT1* target. Garnett’s results provide a partial cross-validation of our predictions, as shown in Table 6.

The ‘drug-specific signaling pathway network’ (DSpathnet)^7^ for metformin generated recently by Sun *et al.* produced results which we reinforce here, although the two studies used different data sets and methodology. The genes that we identified as critical components in our predicted sub-networks overlapped significantly with important components of metformin’s DSpathnet. We compared sub-network nodes which were critical (betweenness-centrality in the 95^th^ percentile for a given sub-network) in a given portion of the sub-networks and were also hubs within the DSpathnet (connectivity >14, n = 38). Seven genes were found to be both critical in at least 25% of sub-networks with a Z-score >1 and were also DSpathnet hubs; they were *BRCA1, TP53, ESR1, STAT1, JUN, SP1*, and *AKT1.* Their work identified seven critical genes (*CDKN1A, ESR1, MAX, MYC, PPARGC1A, STK11*, and *SP1*). All of these genes were identified as critical within at least one predicted sub-network except for *STK11* and *MYC*, which still appeared as nodes within at least one sub-network.

It is interesting to note that the mutations in several genetic biomarkers may lead to the rewiring of the cancer network, as supported by a recent study^26^. We have marked genes on Tables 4 and 5 which were proposed to be involved in this process.

## Discussion

### The putative targets of metformin are largely consistent with existing experimental evidence, suggesting that metformin is a polypharmacological agent with multiple weak binding partners

Our results provide evidence for the direct interaction between metformin and several protein targets whose activities contribute directly to the observed pharmacological effects of the drug. These may be considered the upstream nodes of metformin’s predicted network, and were identified independent of context. Several of these predicted targets and functionally-important network nodes have been previously characterized as related to metformin’s effects because of their presence in pathways which are related to AMPK signaling, such as MAPK signaling and RAS signaling, but here for the first time, we have predicted direct interactions between metformin and these proteins using binding site analysis.

There is strong evidence to suggest that many of our putative targets are both directly and functionally linked to metformin. EGFR is a well-known anti-cancer drug target^27^. Of the kinases we tested for binding with metformin, the strongest direct binding was observed with SGK1. SGK1 participates in many cellular pathways but has gained significant attention in the field of molecular oncology due to its role in several cancer cell pathways^28^. SGK1 is activated by insulin, growth factors, and steroids – this may explain the increased cancer risk for patients with obesity and insulin resistance^29^, as well as explain the dual activity of metformin (which we hypothesize to act as an inhibitor of SGK1) in diabetics as well as cancer patients. Earlier, we mentioned that metformin directly acts on the mitochondria^6^; this result is consistent with our results because SGK1 has been shown to localize in mitochondria in response to cellular stress^30^. It is possible that interaction with SGK1 allows for metformin’s action on mitochondrial respiration. Other studies^28^,^29^ on SGK1 provide evidence of its active role in cancer-related pathways. D’Antona *et al.* using a specific inhibitor of SGK1 kinase (Si113) tested the proliferation and sensitivity to the antineoplastic agent, Paclitaxel, of colon carcinoma cells. Their results indicate that inhibition of SGK1 enhances apoptotic processes and responsiveness to the Paclitaxel drug and also decreases cell viability^29^, which in turn, results in a decrease in the proliferation of the carcinoma. They also claim that, given the success of their inhibitor in affecting colon carcinoma, SGK1 inhibitors are excellent candidates for selectively hitting cancer cell pathways without (or with minimal) interference to normal cell pathways. This is because of the reported over-expression of SGK1 in carcinoma cells relative to normal cells^29^. Wu *et al.* have elucidated some of the purported pathways and mechanisms by which SGK1 may act in the cancer cell lines. The study by Wu *et al.* shows that SGK1 expression inhibits chemotherapy induced apoptosis^28^.

We confirmed binding between metformin and CDK7, a protein involved both in cell cycle control and the activation of RNA-polymerase 2 for transcription. CDK7 phosphorylates many proteins, including p53, a critical regulator of apoptosis and DNA repair^31^. Our putative sub-networks re-constructed the strong functional relationship between CDK7 and both P53 and RNA polymerase-2.

We also demonstrated the binding of metformin and MAPK14. The precise role of MAPK14 depends upon the tumor subtype and there are cases where inhibition, rather than activation, of MAPK14, has anti-cancer effects^32^,^33^,^34^. For example, in one study, inhibition of MAPK14 phosphorylation (accomplished by using the drug Sorafenib), was shown to suppress non-hodgkins lymphoma^32^. A different study on Metformin’s effect on human epidermoid carcinoma, shows a similar decrease in the activation of MAPK14^33^ with a suppressive effect on cancer cell proliferation; another study, shows that Metformin directly decreases the phosphorylation levels of MAPK14 in bovine granulosa cells^35^, a third study shows that Metformin, used in conjunction with gefitinib, decreased levels of MSH2 protein in human lung squamous cell carcinoma, again, through down regulation of MAPK14^34^. Finally, a study on metastatic melanomas shows that activation of MAPK14 by urokinase plasminogen activator receptor (uPAR) clustering on cell surfaces (promoted by d-GM3, a ganglioside) results in the activation of matrix metalloproteinase-2 (MMP-2)^36^ which contributes to cell migration and metastasis in cancer cells. This supports our findings with regards to MAPK14 as a putative target of Metformin, and provides some justification for Metformin’s purported anti-cancer activity. Furthermore, inhibition of MAP2K2 (MEK2), an important signaler in the MAPK signaling pathway, dramatically decreases semaphorin-7a expression in oncogenic Ras cells^37^. We confirmed the binding of MAP2K2 and metformin and predicted the binding of Semaphorin-7a and metformin. Reynolds described the pathway through which our predicted-target Vav1 activates MEK/ERK signaling via Ras^38^.

The NOS3 protein, another one of our predicted targets, catalyzes the synthesis of nitric acid, which facilitates the activation of Ras proteins. Knockdown of NOS3 showed that active NOS3 is necessary both for initiation and maintenance of human pancreatic cancer cells. A study by Reynolds *et al.* suggests that NOS3 acts a link between the different varieties of Ras (HRas, NRas, Kras). It appears that NOS3 activation is increased by the signaling of oncogenic Ras, but the effect of NOS3 comes from subsequent activation of wild-type Ras – for example, oncogenic KRas will lead NOS3 to activate wild-type HRas and NRas^39^.

Allegra demonstrated the role of Semaphorin-7a (SEMA7A) in epithelial to mesenchymal transition (EMT)^37^, an important step in tumorigenesis. The researchers showed that EMT could be prevented by mutating the gene *ERF* so that it could not be phosphorylated. Semaphorin-7a is normally induced by TGF-b, but when *ERF* was mutated Semaphorin-7a was expressed at decreased levels and could not be induced by TGF-b. They next demonstrated that Semaphorin-7a is required for TGF-beta-induced EMT.

The identification of several proteins known to be closely related to metformin’s known activity as direct targets lends credence to the idea that the other identified proteins may also directly interact with metformin, although these interactions may less directly lead to metformin’s anti-cancer activity. We have demonstrated the direct binding between metformin and five out of six candidate proteins, and our predictions and evidence strongly support the notion that metformin is a polypharmacological and pleiotropic drug. Where possible, additional ligand binding assays should be performed between metformin and other predicted targets in order to complete the picture of metformin’s biological activity. Our predictions and experimental results suggest that metformin elicits its strong effects by binding weakly with many targets that act in concert to produce a powerful polypharmacological effect. Similar mechanisms have been proposed for the anti-cancer effect of HIV protease inhibitor Nelfinavir^40^.

### Non-metformin-binding genes could be responsible for the therapeutic effect of metformin

In addition to the direct upstream binders of metformin, the predicted protein sub-networks recapitulate many of the pathways we expected to produce metformin’s anti-cancer activity. We note that our network analysis is only based on one single sample, and is context-specific.

Ouchi *et al.* postulated an “adiponectin-AMPK-PI3-kinase-Akt-eNOS signaling axis” in type 2 diabetes^41^. Our results are consistent with this model, with our predicted binding between metformin and NOS3, and the inclusion of several genes involved in adipocytokine signaling within predicted interaction sub-networks, including the adipokine Leptin and the transcription factors STAT1 and STAT3.

Our predicted sub-networks have several hub genes (Table 4) which may play critical roles in eliciting metformin’s effects. Several hub genes are enriched in higher-scoring sub-networks (Table 5). Many of them have been previously documented to play a role in cancer, and others may be novel cryptic factors specific to metformin’s modulation pathway. They include the *TP53*, *PCNA, and AKT1* genes, which code for the tumor-suppressor p53, the DNA-repairing PCNA protein^42^, and the apoptosis-inhibitor AKT1. We see several cancer-relevant genes which are critical specifically within the most participatory sub-networks. Overexpression of *RAC1* has been noted as a marker for aggressive breast cancer^35^. *STAT1* expression levels have been used as part of a five-gene signature for non-small-cell lung cancer survival^43^. These also include the *BIRC5* inhibitor of apoptosis, as well as the *JUN* and *FOS* genes, coding for c-Jun and c-Fos, which together form the AP-1 transcription factor, and *NFKBIA*, an inhibitor of the survival-promoting NF-κB complex. Huang *et al.* showed that down regulation of c-Jun and NF-κB inhibits the invasion of melanoma cells through suppression of MMP-9^44^ None of our predicted networks included the *MMP9* gene, however the interactions annotated in the STRING-DB are not all direct, and further investigation may identify a role for MMP-9 in metformin’s anti-cancer activity. The growth-promoting *INS-IGF2* gene, and the *SRC* oncogene were both found to be critical in a large percentage of networks, however their critical role appears to be approximately the same in the experimental and control networks. They also are not critical in two of the most highly-participatory networks, based on *MAPK14* and *IL12B*. These genes may still play a critical role in the overall protein-protein interaction network.

Although our networks identified many critical factors which appear promising for experimental validation, it is important to consider the potentially-large portion of false-negatives within our results. For example, our sub-networks were generated with a maximum depth of 5, limiting the resolution of the final network. This allowed us to perform our analyses computationally-efficiently and revealed many strong functional connections. However, it appears that sub-networks generated using randomly-chosen kinase genes as the root (K-control) are enriched for cancer-related nodes at roughly the same rate as sub-networks based on our putative-targets. This suggest that potentially many of these kinases play significant roles in eliciting metformin’s effect beyond what is apparent in the predicted sub-networks.

Network constraints must also be considered when interpreting the significance of peripheral network nodes. A detail from the publication by Sun *et al.* illustrates this point^7^ Their ‘Drug-specific signaling pathway network’ was also applied to metformin. Their methodology attempted to create a complete network of the genes through which metformin elicits its effect, and our work partially recapitulates their results. Their predicted network shares significant overlap with ours; however, they do not speculate on metformin’s direct binding targets. In addition, their network was built using gene expression in response to the metformin treatment from ConnectivityMap (cMAP). While the data is informative, there is also limitation on the direct evidence of those differentially expressed genes in metformin response. Our efforts our distinguished by the fact that our predicted networks are rooted in putative binding targets, some of which we have tested and confirmed.

In metformin’s DSPathNet, the *PPARGC1A* gene serves as a linker node connecting distinct modules of signaling pathways. Variants of this gene have been linked to breast cancer risk^45^ and the gene’s product has been shown to play a key role in the metabolism of ERBB2 + breast cancer cells *in vivo*^46^. The common location for *PPARGC1A* within our sub-networks is as a peripheral node downstream of *TP53*. The motif shows *TP53* as a hub node linked to many genes, including *SIRT1*. *SIRT1* is linked to *PPARGC1A* which connects to *MUM1L1*, a leaf node. The connection between *PPARGC1A* and *MUM1L1* is notably weak, suggesting that the PCST algorithm connects it to our sub-networks with difficulty. It is plausible that *PPARGC1A* may play a larger role in eliciting metformin’s anti-cancer effects than our predictions suggest, owing to the constraining set of differentially expressed genes we used when generating the sub-networks. Future applications of the methodology demonstrated here, may wish to limit the number of false negatives by choosing larger sets of network leaf nodes obtained from multiple sources.

Many of the genes in our predicted sub-networks may play an important role in motivating metformin’s anti-cancer activity. We have identified both upstream direct binding targets for metformin, as well as downstream network nodes which transduce its effects to the level of gene expression. We expect that disruption of the putative targets, network hubs, or nodes which play key roles in high-scoring networks (Tables 3, 4, and 5) is likely to inhibit the effects of metformin or cause adverse side effects.

However, as metformin appears to inhibit cancer broadly and through weak binding with several targets, the specific role played by individual network components in a patient is expected to vary based on the individual genetic and homeostatic context. Further experiments, including gene knock-down experiments, may narrow down the predicted biological role for these proteins, paving the way for their adoption as biomarkers for metformin’s efficacy or adverse drug effects.

### Towards precision medicine using structural systems pharmacology

Data-driven, network-based association studies provide a promising avenue to realize precision medicine^47^. However, the power of data-driven approach remains limited if sample sizes are small. Rare mutations may have unexpected consequences on drug response^48^. To predict drug response *de novo* under diverse genetic backgrounds, a mechanistic understanding of drug action is required. A drug commonly interacts with multiple targets. Each drug-target interaction modifies the conformational dynamics of the target structure and leads to the change of the functional states. Consequently, the changing conformational and functional states of drug-target interactions affect other molecular components and their interactions through the interplay of complex signal transduction, gene regulation, and metabolic networks that collectively mediate the system-level response to the drug, leading to either therapeutic or adverse effect. A variety of genetic/epigenetic alternations, if involved in the drug interaction and modulation, could impact the drug response. Thus, to associate individual genotype to drug response phenotype, it is essential to model drug actions on multiple scales, from the conformational dynamics of genome-wide drug-target interactions to the emergent property of cellular networks, using a structural systems pharmacology approach^49^.

Using metformin as a proof-of-concept study, we demonstrate that structural systems pharmacology is able to provide mechanism-based predictive models using a limited number of samples. The power of structural systems pharmacology depends on the quality and coverage of protein structure, molecular interaction, and gene regulatory network, and molecular phenotypic data. In this study, only AMPKβ and DPP4 are used as templates and ~7000 human protein structures are searched through for the potential targets of metformin. It is likely that many targets of metformin remain to be elucidated. With the exponential increase in experimentally solved structures, it is possible for us to construct high-quality 3D drug-target interaction models on a proteome scale. The molecular details of protein-ligand information will allow us to predict the impact of amino acid mutations on the drug action *de novo*. Moreover, the interaction network applied in this study is incomplete, noisy and is not tissue specific. It may limit the capability to detect novel genetic biomarkers. Recent advance in the proteome-scale mapping of the human interactome network has produced homogenous coverage of the human interactome, significantly expanding the cancer landscape^50^. The ENCODE project has generated genome-wide gene regulatory networks^51^. Coupled with molecular phenomics^52^, we may construct less biased and more complete individual-specific interaction networks^53^,^54^. It is expected that the accuracy in the current network analysis will be significantly increased. Putting these together, structural systems pharmacology could be a powerful tool for precision medicine.

## Materials and Methods

### Overview: a structural systems pharmacology approach to predictive modeling of drug actions under diverse genetic background

As shown in Fig. 2, our methodology begins at the level of molecular structure. We start by ranking the proteins annotated within the PDB by the similarity of their binding sites to that of AMPKβ and dipeptidyl peptidase-IV (DPP4) – two proteins which have been observed to interact directly with metformin^9^,^16^. We considered the top hits from this comparison to be our “putative molecular targets”, and simulated the binding between metformin and each putative molecular target to assess the binding pose and interactions between ligand and target. Next, we moved to the larger and more abstract scale of the protein-protein interaction network. We obtained a dataset which represents genes whose expression is perturbed by metformin treatment. Then, for each putative molecular target, we computationally predicted a tree-shaped path (TSP) which functionally linked that putative molecular target to all genes whose expression is perturbed by metformin. We then analyzed the intermediary nodes of each TSP in terms of their association with biological pathways linked to cancer or AMPK signaling, and identified the most critical nodes of each network. Finally, we experimentally validated the binding between metformin and several putative molecular targets. See Supplemental Methods for further details.

## Additional Information

**How to cite this article**: Hart, T. *et al.* Toward Repurposing Metformin as a Precision Anti-Cancer Therapy Using Structural Systems Pharmacology. *Sci. Rep.* **6**, 20441; doi: 10.1038/srep20441 (2016).

## Supplementary Material

Supplementary Information

Supplementary Datesets

## Figures and Tables

**Table 1:** Putative targets of metformin identified from ligand binding site comparison.

PDB code	Gene symbol	SMAP P-value	Vina binding score Kcal/mol
1Z0M	PRKAB1	0.0	−4.7
1Z68	FAP	1.0E-10	−5.0
3A8N	TIAM1	9.22E-04	−4.8
1SXJ	RFC1	4.79E-03	−5.1
3B2V	EGFR	6.79E-03	−5.1
1S9I	MAP2K2	6.92E-03	−5.1
2BU7	PDK2	9.85E-03	−4.9
3DDU	PREP	1.05E-02	−4.5
3NVQ	SEMA7A	1.63E-02	−4.6
1F45	IL12B	1.71E-02	−5.7
1CJY	PLA2G4A	1.85E-02	−4.7
2ONL	MAPK14	1.96E-02	−5.7
3O96	AKT1	2.49E-02	−5.5
1K8Q	LIPF	2.81E-02	−4.5
3KY9	VAV1	3.01E-02	−6.4
1UA2	CDK7	3.21E-02	−5.5
3HDN	SGK1	3.37E-02	−4.1
2OCI	BPHL	3.50E-02	−5.4
2WWW	MMAA	3.71E-02	−4.6
3EAH	NOS3	3.77E-02	−5.7
1TG6	CLPP	4.05E-02	−2.6

Binding between underlined entries and metformin was experimentally tested by KINOMEscan assay. Four putative targets were identified (PDK2, MAP2K2, EGFR and TIAM1) using AMPKβ as a template, and AMPKβ itself was also included (PRKAB1 gene; no p-value is available due to its use as a template).

**Table 2:** Results of KINOMEscan binding assay between metformin and top-ranked kinase targets.

Gene symbol	% control
SGK1	59
EGFR	60
CDK7	79
MAP2K2	80
MAPK14	88
AKT1	100

‘% control’ is a measure of binding strength. A higher value indicates a weaker interaction; ‘100% control’ indicates no interaction was observed. The results indicate that metformin binds weakly with a large number of kinases.

**Table 3:** Z-score for participation within key pathways.

Root	Z-score
AKT1	2.777
IL12B	2.585
EGFR	2.521
MAPK14	2.265
TIAM1	2.073
SGK1	1.498
RFC1	1.434
SEMA7A	1.306
NOS3	1.115
VAV1	1.051
PLA2G4A	0.923
CLPP	0.859
MMAA	0.795
CDK7	0.667
LIPF	0.411
MAP2K2	0.220
PREP	0.156
FAP	−0.164
PDK2	−0.867
PRKAB1	−1.442
BPHL	−1.570

Calculated by normalizing participation score against the mean of the R-control set. Higher participation indicates that the intermediate nodes of the network are involved in key pathways to a greater extent.

**Table 4:** Genes that were critical nodes in the sub-network.

Critical genes	Number of sub-networks where critical
TP53*	20
AKT1*	20
PCNA*	17
SRC*	15
INS-IGF2	12
SF3A2	10
BIRC5	10
DKC1	9
ESR1*	8
BUB1	7
BRCA1	7
RPL11	7
YBX1*	7
RPS16*	6
GRB2*	6
POLR2H	6
HNRNPK	5
MDM2	5
CASP3	5
POLR2A*	5
CDK5	5
SP1	5

A node is included if the gene appears in at least 20% of experimental sub-networks (5 out of 21). These nodes have a critical role in several sub-networks and may be key cryptic genetic factors in metformin’s signaling network. *indicates genes which have known carcinogenic mutations that could significantly alter the networks.

**Table 5:** Genes where betweenness-centrality was enriched among one sub-group relative to another.

Gene	Control (n = 19)	Experimental (n = 21)	Z > 1 (n = 10)	Z > 2 (n = 5)
SF3A2	37.5%	47.6%	60.0%	100.0%
ESR1*	25.0%	38.1%	50.0%	80.0%
BIRC5	37.5%	47.6%	70.0%	80.0%
MDM2	6.3%	23.8%	30.0%	60.0%
STAT1*	0.0%	14.3%	30.0%	60.0%
POLR2H	18.8%	28.6%	50.0%	60.0%
CTNNB1	6.3%	9.5%	20.0%	40.0%
CREBBP	0.0%	14.3%	30.0%	40.0%
RAC1	0.0%	14.3%	30.0%	40.0%
JUN	6.3%	19.0%	40.0%	40.0%
FOS	6.3%	14.3%	20.0%	40.0%
STAT3	6.3%	19.0%	20.0%	40.0%
IL8	0.0%	9.5%	20.0%	40.0%
NFKBIA*	0.0%	9.5%	20.0%	40.0%
BRCA1	50.0%	33.3%	40.0%	20.0%
HDAC1	37.5%	14.3%	10.0%	0.0%
CDK5	0.0%	23.8%	30.0%	0.0%
SDC2*	18.8%	19.0%	30.0%	0.0%

For example, SF3A2 was considered critical in 37.50% of sub-networks in the control group, but 100% of experimental networks with Z > 2. Shown here are genes where the disparity was at least 25 percentage points between most-enriched and least-enriched. Many of these nodes are enriched in the highest-scoring networks and may be critical nodes specific to metformin’s anti-cancer activity. *indicates genes which have known carcinogenic mutations that could significantly alter the networks

**Table 6:** Genes on the left were independently identified as factors in metformin sensitivity in cancer via the attribute indicated in the central column.

EN-identified gene	Genomic attribute	Putative target(s) or gene(s) from sub-network
*BRCA1*	Copy number	*BRCA1*
*BLM*	Copy number	*BLM*
*AKT3*	Expression	*AKT1*
*IL2RG*	Expression	*IL2*
*MAP4K3*	Expression	*MAP2K2, MAP2K6, MAPKAPK2, MAPK14, MAP3K5, MAPK1*
*RBM15*	Expression	*RBM5*
*RPS6KC1*	Expression	*RPS6, RPS16*
*UBE2G1*	Expression	*UBE2I*

These genes are associated with the factors included on the right, which were part of our set of putative targets or sub-networks.
