# Supplementary material for: Toward Repurposing Metformin as a Precision Anti-Cancer Therapy Using Structural Systems Pharmacology
Source: Sci Rep. 2016 Feb 4;6:20441. doi: 10.1038/srep20441 (PMC4740793; doi:10.1038/srep20441)
Supplement: Supplementary Information [file srep20441-s1.pdf]

Toward Repurposing Metformin as a Precision Anti-Cancer Therapy Using Structural Systems Pharmacology

Thomas Hart[1,2], Shihab Dider[2], Weiwei Han[3], Hua Xu[4], Zhongming Zhao[5,6,7], and Lei Xie[8,9,*]

Author Affiliations

[1]Thomas Hart
The Rockefeller University, New York, New York, United States of America

[2]Thomas Hart and Shihab Dider
Department of Biological Sciences, Hunter College, The City University of New York, New York, New York, United States of America

[3]Weiwei Han
The Key Laboratory for Molecular Enzymology and Engineering, Ministry of Education
Jilin University, Changchun, P. R. China

[4]Hua Xu
School of Biomedical Informatics, The University of Texas Health Science Center at Houston, Houston, Texas, United States of America

[5]Zhongming Zhao
Department of Biomedical Informatics, Vanderbilt University School of Medicine, Nashville, Tennessee, United States of America

[6]Zhongming Zhao
Department of Psychiatry, Vanderbilt University School of Medicine, Nashville, Tennessee, United States of America

[7]Zhongming Zhao
Department of Cancer Biology, Vanderbilt University School of Medicine, Nashville, Tennessee, United States of America

[8]Lei Xie
Ph.D. Program in Computer Science, Biology, and Biochemistry, The Graduate Center, The City University of New York, New York, New York, United States of America

[9]Lei Xie
Department of Computer Science, Hunter College, The City University of New York, New York, New York, United States of America

Supplemental methods

Materials and methods

1. Overview: a structural systems pharmacology approach to predictive modeling of drug actions under diverse genetic background.

Our methodology begins at the level of molecular structure. We start by ranking the proteins annotated within the PDB by the similarity of their binding sites to that of AMPKβ [1] and dipeptidyl peptidase-IV activity (DPP4), a protein which has been observed to interact directly with metformin [2]. We considered the top hits from these comparisons to be our "putative molecular targets", and simulated the binding between metformin and each putative molecular target to assess the binding pose and interactions between ligand and target. Next, we moved to the larger and more abstract scale of the protein-protein interaction network. We obtained a dataset which represents genes whose expression is perturbed by metformin treatment [3]. Then, for each putative molecular target, we computationally predicted a tree-shaped path (TSP) which functionally linked that putative molecular target to all genes whose expression is perturbed by metformin. We then analyzed the intermediate nodes of each TSP in terms of their association with biological pathways linked to cancer or AMPK signaling, and identified the most critical nodes of each network. Subsequently, we experimentally validated the binding between metformin and several putative molecular targets. Finally, we mapped the observed cancer mutations to the binding pockets of experimentally validated targets, and provided clues to the individualized response of metformin.

2. Prediction of molecular targets

Previously, we have constructed 3-dimensional complex models of metformin and AMPKβ and used it to search for the targets of metformin. In addition, DPP4 was retrieved as a molecular target directly binding to metformin by querying the MySql database of ChEMBL release 16. SQL and its output is shown in Figure S1. Recent studies support that metformin inhibits the activity of DPP4 [4 5]. Thus, we used the DPP4 (PDB ID 3O95) as another template for the binding site analysis [6]. The SMAP software developed by Xie et al obtains protein structures from the PDB and characterizes that protein's ligand-binding potential from the geometric, physiochemical, and evolutionary characteristics of its binding pocket. The software compares protein structures and accurately predicts the binding site similarity between the template and all other available structures [7]. Using the crystal structure for AMPKβ bound to metformin and DPP4 bound to TAK-100 as the templates and 7277 available non-redundant PDB structures for human proteins as the test sample, we identified all structures with similar binding sites at a confidence of 95% which were not homologs of DPP4. The p-value of ligand binding site similarity was corrected using the ROC curve of a benchmark set.

We obtained structures for these proteins co-crystallized with their ligand from the PDB, as well as a 3D structure for metformin. Autodock Vina [8] was used to predict the binding pose and energy of binding between each protein and metformin. These reverse-docking experiments were performed at a simulated pH of 7. The size of the binding region was determined by re-docking each protein's original ligand to the pocket and choosing the size which minimized the RMSD

between the location of the original ligand and the re-docked ligand. Binding interactions were analyzed using DS Visualizer [9] and visualized using PyMOL [10].

3. Experimental validation of metformin's interaction with kinases

To test the accuracy of our binding site analysis, we employed a competition binding assay to detect the binding of metformin to a set of kinases chosen from our putative targets. We examined binding between metformin and six top-ranked kinases, namely AKT1, SGK, CDK7, and MAPK14, MAP2K2, and EGFR. The proprietary KINOMEscan assay was performed by DiscoverX (CA). The assay tested the capacity for metformin to disrupt the binding of each DNA-tagged kinase to a support which was in turn bound to the kinase's known ligand. If binding between the kinase and its known ligand was disrupted in the presence of metformin, this indicated that metformin either competed directly with the known ligand or allosterically altered the kinase's ability to bind to that ligand. DMSO was used as a positive control and a pico-molar kinase inhibitor was used as a negative control. Binding levels were quantified by performing qPCR on the DNA tag of the ligand-bound kinases. The tests were performed at $1x10^6$ nM concentration of metformin, and results were reported as %Control, calculated as follows:

$$\frac{(test\ compound\ signal - positive\ control\ signal)}{(negative\ control\ signal - positive\ control\ signal)}\ X\ 100$$

A lower %Control score indicates a stronger interaction. The KINOMEScan experiment and data analysis were performed by DiscoveRx (Fremont, CA).

4. Identification of drug-modulated interaction sub-networks

We obtained human protein-protein interaction data from STRING-DB [11], and converted the Ensembl-protein IDs [12] to Unigene IDs [13], and then to official gene symbols [14] [15]. We removed entries for two ubiquitin genes, *UBC* and *UBA52,* from the network to prevent their high degree of connectivity from introducing bias into our predicted sub-networks.

The terminal nodes for our network were chosen from a set of genes which were differentially-translated in MCF7 breast cancer cells under metformin treatment, as published by Larsson et al [3]. By microarray quantification of mRNAs from cytoplasmic and polysomal samples, the group found that the primary effect of metformin on gene expression is the inhibition of translation. Their Analysis of Translational Activity methodology identified transcripts with the absolute value of fold change > 1.5 and Benjamini-Hochberg false-discovery rate < 0.15 as differentially translated. Supplementary Document 2 of their publication contains the set of differentially-translated transcripts mapped to specific genes, 213 of which were differentially-translated by metformin at its absolute IC50 concentration of 10mM. Of these, 196 mapped to nodes within our protein-protein interaction network and were considered the leaf (terminal) nodes. For cases where a predicted molecular target was found amongst the down-regulated genes, the gene was removed from the list of down-regulated genes for that analysis. The differentially-expressed genes from this set used in our analysis are re-produced in Supplemental Data 1 with their log2 fold change and FDR value as originally published by Larsson et al.

Using the MSGSTEINER software, we generated a protein-protein interaction sub-network for each metformin-interacting protein [16]. The parameters used in this study are shown in Table S2.

Each sub-network represents the most parsimonious series of interactions found within the STRING-DB that connects each root node to all differentially-translated genes. Edges were weighted as the strength of the protein-protein interaction as documented in the STRING-DB, normalized to a score between zero and one. As our comparison of binding site experiment initially yielded 16 putative targets, as a control we also generated interaction networks using the same terminal nodes for 16 randomly-selected genes (R-control) whose protein products were annotated within the STRING-DB [17]. A second control set was produced using 20 genes selected at random from the set of leaf nodes (L-control). A third control set was generated using a random selection of 19 human genes (K-control) which were annotated with the 'kinase activity' Gene Ontology term (GO:0016301).

A previous study tested the possibility that metformin's effects are elicited through direct interaction with AMPK. The PDB structure for the AMPK subunit PRKAB1 (PDB ID 1Z0M) was used as a template for binding site comparison, and the structures for MAP2K2 (PDB ID 1S9I), EGFR (PDB ID 3B2V), TIAM1 (PDB ID 3A8N), and PDK2 (PDB ID 2BU7) were identified [1]. Additional sub-networks were generated using these five putative targets as roots.

5. Analysis of interaction sub-networks

With the goal of differentiating between the predicted content of each sub-network, we removed the terminal nodes from the gene set for our interaction networks. We used the online tool GeneTrail to perform over- or under-representation analysis of these gene sets to identify biological pathways relevant to metformin's putative mechanism of action or other cancer-related pathways [18]. The significance threshold was set to 0.05 and the false discovery rate was controlled by the Benjamini-Hochberg method, and the minimum number of genes that must be part of a pathway for it to be considered significant was set to 2.

The following KEGG pathways were defined as 'cancer-related' from among the pathways identified as enriched by GeneTrail in at least one sub-network: 'Prostate cancer', 'Glioma', 'Chronic myeloid leukemia', 'Pancreatic cancer', 'Non-small cell lung cancer', 'Bladder cancer', 'Small cell lung cancer', 'Acute myeloid leukemia', 'Colorectal cancer', 'Endometrial cancer', 'Melanoma', 'Renal cell carcinoma', 'Thyroid cancer', 'Nucleotide excision repair', 'ErbB signaling pathway', 'MAPK signaling pathway', 'DNA replication', 'Apoptosis', 'Cell cycle', 'Pathways in cancer', 'p53 signaling pathway', 'VEGF signaling pathway', 'mTOR signaling pathway', 'Homologous recombination', 'Mismatch repair', 'TGF-beta signaling pathway', 'Chemokine signaling pathway', 'Jak-STAT signaling pathway', 'Wnt signaling pathway', 'Base excision repair', and 'Cytokine-cytokine receptor interaction'.

Based on the results of the pathway analysis, we chose several key KEGG pathways to examine. These pathways included cancer-related pathways as well as pathways linked to metformin's demonstrated regulation of metabolism and AMPK signaling: 'AMPK signaling', 'MAPK signaling', 'ErbB signaling', 'mTOR signaling', 'B cell receptor signaling', 'Adipocytokine

signaling', 'Neurotrophin signaling', 'Insulin signaling', 'VEGF signaling', 'DNA replication', 'Apoptosis', 'Cell cycle', 'Pathways in cancer', and 'Nucleotide excision repair' [19]. We evaluated the number of genes from each interaction network that participated in each of these pathways. Both terminal and constituent nodes were included in this analysis.

We used the Markov Cluster algorithm to rank the nodes of these sub-networks by their betweenness-centrality in an attempt to determine which genes are most critical to the functionality of the interaction network [20 21]. We defined a node as critical if its betweenness-centrality value was ranked in the 95th percentile for the sub-network in question. We identified those nodes that are critical across all predicted sub-networks, as well as those that were grouped within highly-participatory sub-networks. We characterized individual genes using the GeneCards database (http://www.genecards.org/).

6. Mutation analysis

For experimentally validated kinase targets of metformin, the binding pose of metformin was predicted using protein-ligand docking software Autodock Vina [8]. The amino acid residues that interact with metformin were determined using DS Visualizer. The mutations observed in COSMIC [22] were mapped to the binding site residues, and visualized using DS Visualizer. The mutations that might lead to the rewiring of protein-protein interaction network were extracted from AlQuraishi et al [23].

Supplemental Figures

```
SELECT td.accession Uniprot, ac.standard_value Value, ac.standard_units Unit, td.pref_name Protein
FROM molecule_dictionary md
INNER JOIN activities ac ON md.molregno=ac.molregno
INNER JOIN assays at ON ac.assay_id=at.assay_id
INNER JOIN single_protein_target_view td ON at.tid=td.tid
WHERE md.molecule_type='small molecule' AND td.accession is not null AND
        at.confidence_score>=8 AND ac.standard_value is not null AND
        ac.standard_type='IC50' AND md.chembl_id='CHEMBL1431';
```

Uniprot	Value	Unit	Protein
P27487	29000.0	nM	Dipeptidyl peptidase IV
O15245	2010000.0	nM	Solute carrier family 22 member 1
O15244	1700000.0	nM	Solute carrier family 22 member 2

Figure S1. SQL query and its output to retrieval direct molecular targets of metformin from ChEMBL release 16. Solute carrier protein is mainly involved in pharmacokinetics of metformin, and may not play roles in its mode of actions. Thus, DPP4 remains as a target.

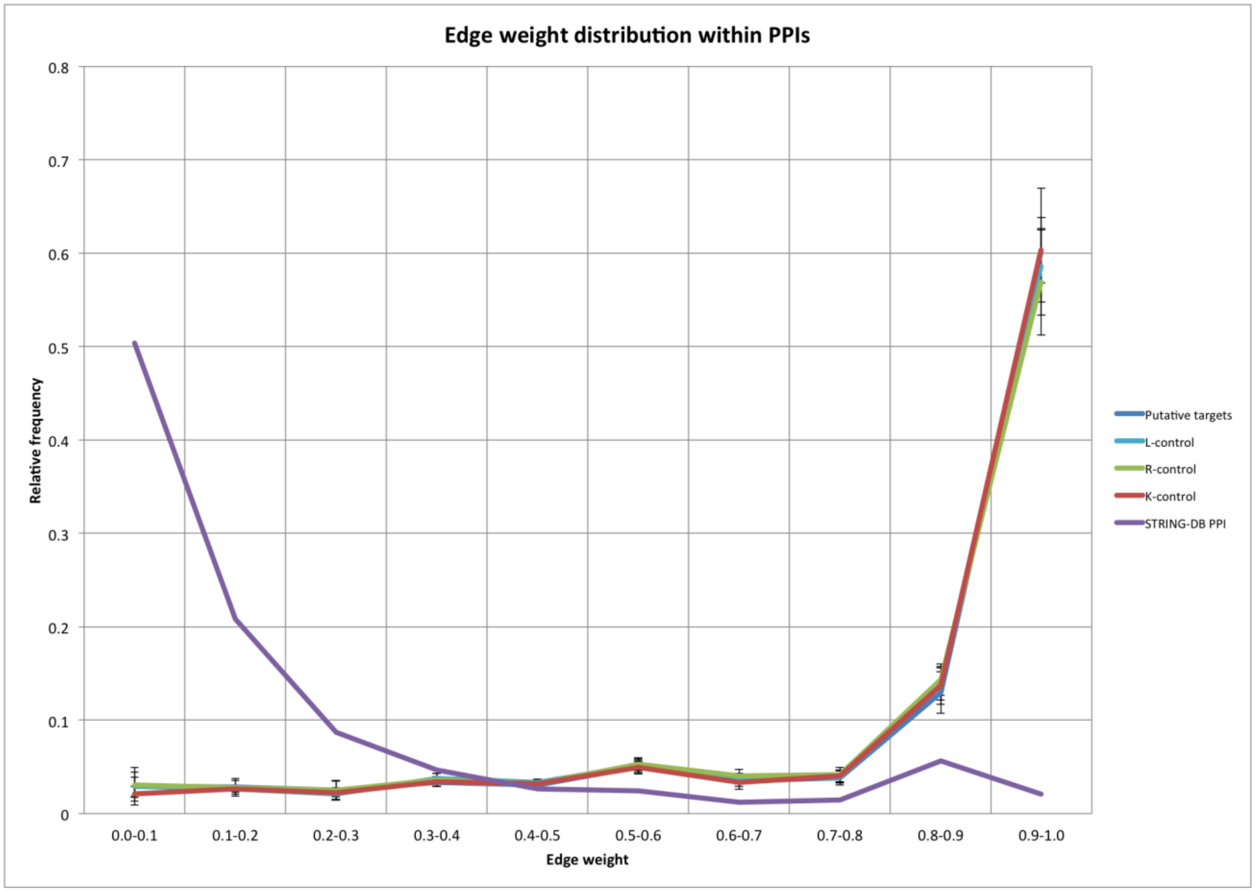

Figure S2: Distribution of edges by confidence in sub-networks and human PPI. Edges were grouped by confidence with an increment of 0.1 on a scale from 0.0 to 1.0. “STRING-DB PPI” includes all interactions documented in humans. The majority of the edges incorporated into sub-networks by the PCST algorithm are of consistently high-confidence, in contrast to the mainly low-confidence edges present in the STRING-DB PPI.

Supplemental Tables

Table S1: Mutations from the COSMIC database which likely affect metformin binding.

Italicized mutations are those that affect residues identified as the binding site on PDB. * indicates mutations which involve charged residues.

MAP2K2 (1S9I)	CDK7 (1UA2)	MAPK14 (2ONL)	SGK1 (3HDN)	EGFR (3B2V)
p.M150L	*p.K41N**	p.R186K*	p.T239N	*p.V323I*
p.D151D (silent)	p.A159D*		p.F241F (silent)	*p.R324H**
*p.Q157H (site AC3)**	*p.S161Y*			*p.R324L**
				*p.C326R**
				p.C326Y
				p.C326S
				p.C329C
				p.I351V
				p.T354M
				p.T354T (silent)

Table S2: Parameters for MSGSTEINER software

Parameter	Value
Terminal node weight	$1x10^{5}$
Intermediate node weight	0
Edge weight	0-1
Reinforcement parameter	$1x10^{-4}$
Convergence tolerance	$1x10^{-5}$
Random factor	$1x10^{-5}$
Maximum depth	5

Supplemental Dataset 1: Set of leaf nodes (differentially-expressed genes). Lines follow the format '[GeneSymbol] [log2 Fold Change] [BH-adjusted FDR]' and are separated by commas. Obtained from Larsson et al.

Supplemental Dataset 2-22: Sub-networks generated with putative targets as root nodes. Lines follow the format '[GeneSymbol 1] [GeneSymbol 2] [interaction confidence]' and are separated by commas. The networks may be visualized using the free CytoScape software [24].

Bibliography

1. Han, W. & Xie, L., *Structural basis of pharmacological effects of metformin*, presented at IEEE International Conference, Philadelphia, 2012 (unpublished).
2. Bento, A. P. *et al.*, The ChEMBL bioactivity database: an update. *Nucleic Acids Res.* **42**, DOI: 10.1093/nar/gkt1031 (2014).
3. Larsson, O. *et al.*, Distinct Perturbation of the Translatome by the Antidiabetic Drug Metformin. *Proc. Natl. Acad. Sci.* **109** (23), 8977-8982 (2012).
4. Lindsay, J. R. *et al.*, Inhibition of dipeptidyl peptidase IV activity by oral metformin in Type 2 diabetes. *Diabet. Med.* **22** (5), 654-657 (2005).
5. Cuthbertson, J., Patterson, S., O'Harte, F. P. & Bell, P. M., Investigation of the effect of oral metformin on dipeptidylpeptidase-4 (DPP-4) activity in Type 2 diabetes.. *Diabet. Med.* **26** (6), 649-654 (2009).
6. Taldone, T., Zito, S. W. & Talele, T. T., Inhibition of Dipeptidyl Peptidase-IV (DPP-IV) by Atorvastatin. *Bioorg. Med. Chem. Lett.* **18** (2), 479–484 (2008).
7. Ren, J., Xie, L., Li, W. W. & Bourne., P. E., SMAP-WS: a Parallel Web Service for Structural Proteome-Wide Ligand-Binding Site Comparison. *Nucleic Acids Res.* **38** (Web Server Issue), W441-444 (2010).
8. Trott, O. & Olson, A. J., AutoDock Vina: improving the speed and accuracy of docking with a new scoring function, efficient optimization and multithreading. *J. Comput. Chem.* **31** (2), 455-461 (2010).
9. Dassault Systèmes BIOVIA, in *Discovery Studio Modeling Environment, release 4.5* (Dassault Systèmes, San Diego, 2015).
10. in *The PyMOL Molecular Graphics System, Version 1.7.4* (Schrodinger, LLC).
11. Jensen, L. J. *et al.*, STRING 8--a Global View on Proteins and Their Functional Interactions in 630 Organisms. *Nucleic Acids Res.* **37** (Database Issue), D412–416 (2009).
12. Flicek, P. *et al.*, Ensembl 2014. *Nucleic Acids Res.* **42**, doi: 10.1093/nar/gkt1196 (2014).
13. McEntyre, J., Ostell, J., Pontius, J. U., Wagner, L. & Schuler, G. D., UniGene: a unified view of the transcriptome. *The NCBI Handbook* (2003).
14. Huang, D. W., Sherman, B. T. & Lempicki., R. A., Systematic and integrative analysis of large gene lists using DAVID Bioinformatics Resources. *Nat. Protoc.* **4** (1), 44-57 (2009).
15. Huang, D. W., Sherman, B. T. & Lempicki., R. A., Bioinformatics enrichment tools: paths toward the comprehensive functional analysis of large gene lists. *Nucleic Acids Res.* **37** (1), 1-13 (2009).
16. Bailly-Bechet, M. *et al.*, Finding Undetected Protein Associations in Cell Signaling by Belief Propagation. *Prot. Natl. Acad. Sci.* **108** (2), 882-887 (2010).
17. Morgane, T.-C. M. *et al.*, RSAT 2011: Regulatory Sequence Analysis Tools. *Nucleic Acids Res.* **39** (Web Server Issue), W86-W91 (2011).
18. Backes, C. A. K. *et al.*, GeneTrail - advanced gene set enrichment analysis. *Nucleic Acids Res.* **35** (suppl 2), W186-W192 (2007).
19. Kanehisa, M., Goto, S., Kawashima, S., Okuno, Y. & Hattori, M., The KEGG resource for

deciphering the genome. *Nucleic Acids Res.* **32** (suppl 1), D277-D280 (2004).
20. Van Dongen, S., in *Graph Clustering by Flow Simulation* (University of Utrecht, 2000).
21. Enright, A. J., Van Dongen, S. & Ouzounis, C. A., An efficient algorithm for large-scale detection of protein families. *Nucleic Acids Res.* **30** (7), 1575-1584 (2002).
22. Forbes, S. A. *et al.*, COSMIC: exploring the world's knowledge of somatic mutations in human cancer.. *Nucleic Acids. Res.* **43** (Database Issue), D805-D811 (2015).
23. AlQuraishi, M., Koytiger, G., Jenney, A., MacBeath, G. & Sorger, P. K., A multiscale statistical mechanical framework integrates biophysical and genomic data to assemble cancer networks. *Nat. Genet.* **46** (12), 1363-1371 (2014).
24. Shannon, P. *et al.*, Cytoscape: a software environment for integrated models of biomolecular interaction networks. *Genome Res.* **13** (11), 2498-2504 (2003).
